# Capacity building in porous materials research for sustainable energy applications

**DOI:** 10.1098/rsfs.2023.0067

**Published:** 2024-08-09

**Authors:** Henrietta W. Langmi, Nicholas M. Musyoka, Justin C. Kemmegne-Mbouguen, Chrispin Kowenje, Fredrick Kengara, Robert Mokaya

**Affiliations:** ^1^ Department of Chemistry, University of Pretoria, Private Bag X20, Hatfield 0028, South Africa; ^2^ Nottingham Ningbo China Beacons of Excellence Research and Innovation Institute, University of Nottingham Ningbo China, Ningbo 315100, People’s Republic of China; ^3^ Faculty of Science, Laboratory of Nanomaterial for Sensors and Energy, University of Yaounde I, Yaoundé B.P. 812, Cameroon; ^4^ Department of Chemistry, Maseno University, P.O. Box, 333-40105, Maseno, Kenya; ^5^ School of Pure and Applied Sciences, Bomet University College, P.O. Box 701-20400, Bomet, Kenya; ^6^ School of Chemistry, University of Nottingham, University Park, Nottingham NG7 2RD, UK

**Keywords:** capacity, porous, materials, research, sustainable, energy

## Abstract

The project aimed to develop porous materials for sustainable energy applications, namely, hydrogen storage, and valorization of biomass to renewable fuels. At the core of the project was a training programme for Africa-based researchers in (i) the exploitation of renewable locally available raw materials; (ii) the use of advanced state-of-the-art techniques for the design and synthesis of porous materials (zeolites and metal-organic frameworks (MOFs)) for energy storage; and (iii) the valorization of sustainable low-value feedstock to renewable fuels. We found that compaction of the UiO-66 MOF at high pressure improves volumetric hydrogen storage capacity without any loss in gravimetric uptake, and experimentally demonstrated the temperature-dependent dynamic behaviour of UiO-66, which allowed us to propose an activation temperature of ≤ 150°C for UiO-66. Co-pelletization was used to fabricate UiO-66/nanofibre monoliths as hierarchical porous materials with enhanced usable (i.e. deliverable) hydrogen storage capacity. We clarified the use of naturally occurring kaolin as a source of silica and alumina species for zeolite synthesis. The kaolin-derived zeolite X was successfully used as a catalyst for the transesterification of *Jatropha curcas* oil (from non-edible biomass) to biodiesel. We also prepared porous composites (i.e. carbon/UiO-66, organoclay/UiO-66 and zeolite/carbon) that were successfully applied in electrochemical sensing.

## 1. Introduction

Energy production processes that do not release greenhouse gases are essential for the development of efficient and sustainable energy supplies. Such processes can be achieved by using energy carriers that remove or reduce CO_2_ emissions. In this regard, hydrogen is widely regarded as a most promising alternative to fossil fuels; it is lightweight, highly abundant (hydrogen is the most abundant element in the universe) and when used as an energy carrier generates no emissions, whether by combustion or in a fuel cell, other than benign water vapour. An energy economy based on sustainable hydrogen*—the sustainable hydrogen energy economy*—has long been viewed as the ultimate goal in renewable or ‘new’ energies. There is, however, a major technological bottleneck due to a lack of suitable solid-state materials that can capture and release hydrogen in sufficient quantities. It is widely accepted that the development of a viable solid-state storage material would lead to a step-change in the transition to a hydrogen energy economy. The scientific challenge is to push the state-of-the-art beyond current knowledge and to prepare materials with ‘made to measure’ properties that will store hydrogen reversibly, cheaply, effectively and efficiently. We aimed to develop and demonstrate the effectiveness of the next generation of nanostructured materials that would enable the storage and end-use of hydrogen as a sustainable energy source for stationary and portable applications. The research required expertise and training (including capacity building and/or strengthening) on porous materials fabrication, characterization and evaluation for renewable energy applications. The development of porous materials with the capacity to store sufficient hydrogen to accomplish the targets required for stationary and portable applications is a challenge. During the past decade a great variety of porous materials, namely, metal-organic frameworks (MOFs), activated carbons, zeolite-like carbon materials and carbide-derived carbons, have been investigated. The current targets require the development of hydrogen storage materials with a capacity of at least ≥ 5.5 wt%, and ≥ 40 kg H_2_ m^−3^, but which can reversibly release the hydrogen at operating temperatures compatible with envisaged application routes, along with appropriate hydrogen loading and unloading kinetics. There are also several other important targets apart from volume, weight and cost, such as hydrogen charging/discharging rates, safety, durability and operability at safe pressures and temperatures. At the present time, there is no material that fulfils all the above-mentioned requirements. The key target of the proposed research was to develop nanostructured/functionalized materials with properties that are tailored to address the limitations of currently available hydrogen stores. This required advances in the fabrication of nanostructured materials so as to enable control of: (i) textural properties (surface area, pore volume and pore size); (ii) ease of preparation; and (iii) choice of precursors.

On the other hand, the catalytic conversion of renewable biomass into biofuels and other energy-relevant chemicals is now recognized as a viable valorization process that can significantly contribute to a reduction of our reliance on fossil-derived fuels. The process of converting low-value biomass into energy carrier chemicals is, however, a technological challenge, as it typically requires a catalyst capable of accomplishing the transformation under aqueous and richly oxygenated operating conditions. In the second strand of our investigations, the project aimed to specifically target a major bottleneck in biofuel production, namely, the high cost of raw materials by employing low-value feed stocks such as non-edible oils from Jatropha*—Jatropha curcas* (JC). The high oil content of the Jatropha seeds, which is indigenous to Kenya, combined with the properties of the plant makes Jatropha oil an easily available and promising resource. As catalysts, the project explored the use of zeolites prepared from modified naturally available (Kenya and Cameroon) silica-based materials as precursors

The consortium comprised four research teams that were well matched to the range of energy materials and processes to be investigated, and to the skills required to successfully carry out the project in terms of research output, training and capacity building. The project offered an opportunity for the synergistic development of novel materials for renewable energy applications that are relevant to both the UK and African partners. Our capacity strengthening/building plan was guided by the need to engender, in African partners, environments that encourage the pursuance of excellent research. At the outset, the consortium endeavoured to harness local expertise in African partners to positively add to the capacity strengthening. The consortium recognized that the three African partners were at different levels with respect to local expertise in energy materials research. In particular, it was important to identify opportunities for African–African capacity-building initiatives between the Council for Scientific and Industrial Research (CSIR), Maseno University and University of Yaounde I, which enhanced already existing best practice. All students registered at African partner universities were expected to spend at least six months in Nottingham. The consortium was set up with the express aim of delivering a comprehensive PhD training programme and innovative research. The intertwined training and research programmes aimed to achieve clearly defined goals, namely, (i) establish research capacity in porous energy materials in the African partner institutions such that they are internationally recognized centres for renewable energy and sustainable research; (ii) develop and embed graduate training schemes that directly feed into capacity building in the African partners; and (iii) train other levels of personnel, namely, technicians.

## 2. Scientific highlights

### 2.1. Metal-organic frameworks for hydrogen storage

The first prong of our work explored the use of MOFs as hydrogen storage materials. MOFs exhibit high surface area, large pore volume and tuneable pore size, meaning that they are potentially viable materials for cryogenic hydrogen storage. At cryogenic temperatures, the adsorption mechanism in porous materials such as MOFs is based on physisorption, which allows for complete reversibility and fast kinetics of hydrogen uptake and release. Despite their promise, the viable and practical use of MOFs in hydrogen storage is still yet to be realized. MOFs are normally synthesized as powders. Therefore, for integration in a hydrogen storage system, it is important to process the MOFs into shaped forms for ease of handling and to enhance other properties such as packing density and, consequently, the volumetric hydrogen storage capacity [1]. Our work focussed on the fabrication of MOFs, specifically the zirconium-based MOF, UiO-66, with the aim of improving the hydrogen storage capacity both on a gravimetric and volumetric basis.

**Figure 1.**
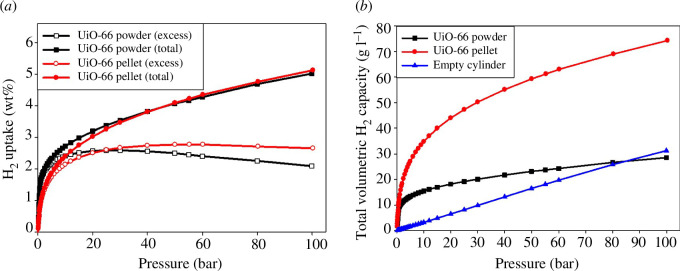
(*a*) Gravimetric H_2_ uptake (wt%) for UiO-66 powder and UiO-66 pellet at 77 K and up to 100 bar. (*b*) Volumetric H_2_ uptake for UiO-66 powder, UiO-66 pellet and an empty cylinder (i.e. contained no adsorbent) at 77 K and up to 100 bar [2].

Following our MOF investigations, we reported on unusual and insightful findings, which elaborated on the effect of compacting UiO-66 at a high pressure of 700 MPa [2]. Compaction enhanced the density of the UiO-66, and consequently the volumetric hydrogen storage capacity. Interestingly, the compaction did not affect the gravimetric hydrogen uptake capacity, which remained similar to that of the powder form of UiO-66 (figure 1). The total gravimetric hydrogen storage capacity at 100 bar and 77 K was 5.1 and 5.0 wt% for the pellet (i.e. compacted form) and powder forms, respectively. The compacted UiO-66 maintained 98% of its initial (i.e. powder form) textural properties, which explained the similar gravimetric hydrogen uptake capacity for the powder and pellet forms. It was further revealed that the volumetric hydrogen storage capacity of the compacted UiO-66 was remarkably high reaching 74 g l^−1^ at 77 K and 100 bar (compared to 29 g l^−1^ for the UiO-66 powder) when calculated using the packing density of the compacted MOF. The packing density increased from 0.57 g cm^−3^ for the UiO-66 powder to 1.45 g cm^−3^ for the compacted MOF. Overall, this aspect of our work demonstrated that MOFs such as UiO-66 can be compacted at high pressure in a manner that improves their volumetric hydrogen storage capacity without compromising their gravimetric hydrogen uptake [2].

**Figure 2.**
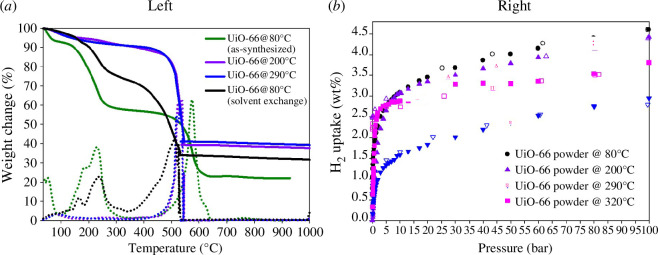
(*a*) Thermal decomposition of as-synthesized UiO-66 and after post-synthesis activation: degassed at 80°C (black); 200°C (purple); 290°C (blue); solvent exchanged with acetone (green). (*b*) Total gravimetric H_2_ uptake isotherms at 77 K for UiO-66 degassed at 80, 200, 290 and 320°C. Open symbols represent H_2_ desorption isotherms [6].

UiO-66 is known to occur in two stable forms, i.e. the hydroxylated form (containing Zr_6_O_4_(OH)_4_ clusters) and dehydroxylated form (containing distorted Zr_6_O_6_ clusters), whereby dehydroxylation is fully achieved at *ca* 300°C [3]. Computationally, it has been revealed that dehydroxylation affects the mechanical stability of UiO-66 [4,5]. In this regard, we reported, for the first time, the experimental demonstration of the unusual observation of the dynamic behaviour of UiO-66 stimulated by changes in the activation temperature [6]. Our work showed that dehydroxylation of UiO-66 occurred at 150–300°C (figure 2). At 100 bar and 77 K, the gravimetric hydrogen storage capacity of hydroxylated UiO-66 powder was 21% greater than that of the dehydroxylated UiO-66 powder. After compaction at 700 MPa, the gravimetric hydrogen uptake capacity of the hydroxylated pellets decreased by 9% whereas that of the dehydroxylated pellets decreased considerably by 66%. Our studies further demonstrated that the hydrogen storage capacity was highly correlated with changes in the porosity of the MOF induced by compaction. We revealed that the porosity, and consequently hydrogen uptake capacity, of UiO-66 was particularly responsive to activation at high temperature. While hydroxylated UiO-66 retained its structural properties and hydrogen uptake capacity after compaction into pellets, the structure of the dehydroxylated UiO-66 was, on the other hand, significantly degraded. We, therefore, proposed that the optimum post-synthesis activation temperature for UiO-66 should not be higher than 150°C. Activation at or below 150°C retains the hydroxylated form, which is suited for enhanced hydrogen storage [6].

One of the most important parameters for practical hydrogen storage is the usable capacity, defined as the amount of hydrogen released between full tank conditions and depleted tank conditions [7]. Strategies to improve the gravimetric and volumetric hydrogen storage capacities, as well as enhance the usable capacity of nanoporous materials were recently presented in a review paper [7]. Taking advantage of the robustness of hydroxylated UiO-66, we combined it with electrospun nanofibres using a co-pelletization approach to fabricate UiO-66/nanofibre monoliths [8]. We found that at least 85% of the UiO-66 powder and pellet porosity was maintained in the co-pelletized UiO-66/nanofibre monoliths. In addition, UiO-66 powder and pellets were predominantly microporous whereas the UiO-66/nanofibre monoliths exhibited a mixture of microporosity and mesoporosity. This resulted in the UiO-66/nanofibre monoliths exhibiting enhanced usable hydrogen storage capacities over those of the UiO-66 powder and pellets. Our work introduced a facile approach, i.e. co-pelletization, to create a hierarchical porous structure in a MOF that is primarily microporous, thus leading to significant improvements in the usable hydrogen storage capacity [8].

### 2.2. Clay-derived zeolites for biodiesel production from *Jatropha curcas*

Clays are layered aluminosilicates that are naturally available or can be synthesized and have interesting properties including large surface area, thermal, chemical and mechanical stability and ion-exchange capacity. Due to these properties, and depending on their layered structure, clays have been used in various applications in agriculture, environmental protection, catalysis and electrochemistry. Clays mainly consist of tetrahedral and octahedral sheets in either 1 : 1 or 2 : 1 configurations. The properties of clays are to some extent dependent on where they occur. In this regard, Cameroon, in West Africa, has large natural deposits of kaolin containing kaolinite (a 1 : 1 configuration clay) and smectite montmorillonite (a 2 : 1 configuration clay). Kaolin has attracted considerable attention as a starting material for the green synthesis of zeolites in which it acts as the natural source of silica and alumina. Zeolites are aluminosilicates characterized by three-dimensional and four-junction framework formed by an arrangement of SiO_4_ and AlO_4_ tetrahedra linked together by their oxygen atoms. Zeolites have a large range of applications including in energy (heat) storage, gas separation and sorption, catalysis (e.g. catalytic transformation of oil or biomass to biodiesel), and more recently as templates in the synthesis of higher forms of porous materials such as porous carbons that are suitable for energy storage.

**Figure 3.**
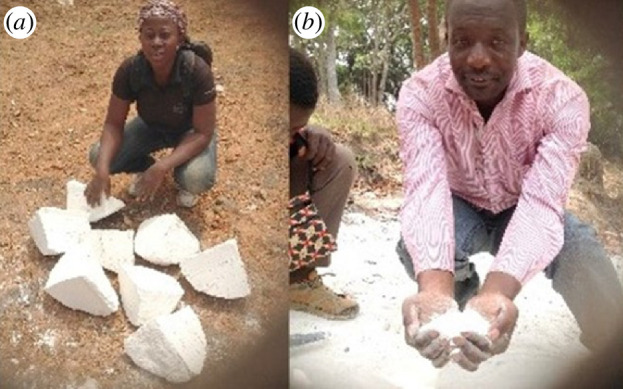
University of Yaounde I PhD student (*a*) and Principal Investigator (PI) (*b*) engaged in kaolin sampling at the Mayouom deposit in Cameroon.

**Figure 4.**
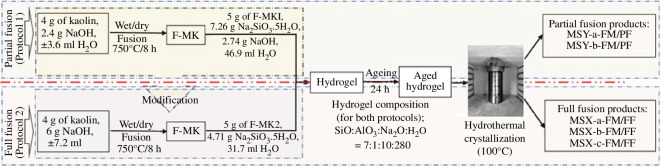
The pre-treatment methods of the materials used for zeolite synthesis [9].

In the second prong of our project, zeolites were derived from quartz-rich kaolin obtained from Mayouom in West Cameroon (figure 3) [9]. The effect of kaolin pre-treatment and the amount of NaOH used in such treatment on the properties of the synthesized zeolites was explored [9,10]. The pre-treatment involved fusion processes that transformed the kaolin into soluble aluminosilicates. The use of NaOH for fusion is known to be effective in the transformation of kaolin to soluble aluminosilicates. However, prior to our work, there was no clarity on how the fusion process influences the properties of the resulting kaolin-derived zeolites. Thus, we clarified the effect of activation with NaOH during the kaolin fusion step of the preparation of kaolin-derived zeolites via either partial or full fusion of the kaolin followed by hydrothermal crystallization. Following the fusion step, we maintained identical subsequent hydrothermal synthesis conditions by adjusting the amount of NaOH to retain a similar hydrogel composition. The partial and full fusion resulted in zeolite Y and zeolite X structures, respectively. As shown in figure 4, for full fusion, dry mixing before dry fusion of kaolin mainly resulted in zeolite X with some zeolite A impurity. Homogeneity in the hydrogel in a manner that avoided the formation of the side-product zeolite A was achieved by wet mixing. Interestingly, dry fusion after wet mixing generated pure zeolite X with high porosity (surface area of 645 m^2^ g^−1^ and pore volume of 0.24 cm^3^ g^−1^), which is comparable to that of commercially available zeolite 13×. This aspect of our work demonstrated how locally available and low-cost materials may be used to generate porous materials (in this case zeolites) with properties comparable to commercially available variants.

**Figure 5.**
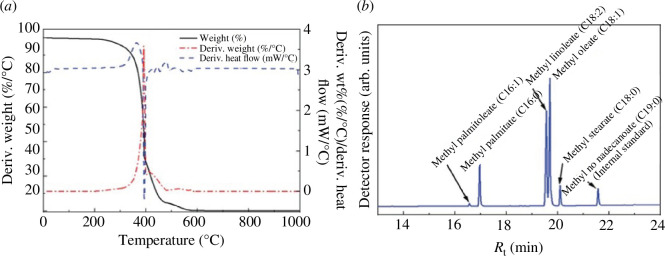
Characterization of JC oil and transesterification products: (*a*) TGA curve of JC oil and (*b*) GC spectra of the biodiesel product [12].

In further demonstration of the use of locally available materials, tropical volcanic ash sampled from Ol Doinyo Eburru, a volcanic mountain in Kenya, was explored as a cheap and sustainable source of pure silica for the synthesis of zeolites. It was noted that the properties of the synthesized zeolites was determined by the amount of SiO_2_ and Na_2_O in the synthesis hydrogel [11]. Importantly, the synthesized zeolites were used as heterogeneous catalysts in biodiesel production. It is worth noting that for conventional biodiesel production, the biomass feedstock used remains the main production barrier as it is competitively used as human food meaning that it has high associated costs. A further challenge is the use of homogeneous catalysts, which pose difficulties in product purification and lead to environmental pollution when disposed. In our work, the main application of the synthesized volcanic-ash derived Na–X zeolite was in the transesterification of non-edible JC oil to biodiesel [12]. The temperature, reaction time, catalyst loading and methanol-to-oil molar ratio were the optimized parameters, using the L16 Taguchi orthogonal array approach. At 93.79%, the catalyst loading was the most influential parameter. A reaction temperature of 70°C, methanol-to-oil molar ratio of 10 : 1, reaction time of 5 h and catalyst (i.e. zeolite) loading of 8% were the optimum conditions giving a 93.94% biodiesel yield from the JC oil. Other biodiesel characterization parameters conformed to European Norm 14214:2019 biodiesel specifications as shown in figure 5.

### 2.3. Porous material composites for electrochemical sensing

Due to the interesting structural properties of carbon/UiO-66 material prepared for hydrogen storage in the consortium’s research, sensitive and selective electrochemical sensors were fabricated based on this composite [13]. Exploration of these composite materials, which formed the third prong of our project work, resulted in the synthesis of zeolite/carbon composites for sensing and energy production. We reported zeolite A and zeolite X with large surface areas synthesized from pre-treated natural kaolin [14]. These zeolites were successfully used to modify carbon paste electrodes for electrochemical sensing. In addition, organo-functionalized clays (type 2 : 1) were prepared and the obtained organoclays were used for the synthesis of two organoclay/UiO-66 composites exhibiting large surface area and electrocatalytic properties when used as electrode modifiers [15,16].

## 3. Capacity strengthening

### 3.1. Training workshops

#### 3.1.1. International workshops

The South African partners hosted several successful international workshops throughout the project. These workshops targeted master’s and PhD students, postdoctoral fellows and early- to mid-career researchers. The first workshop was held as a pre-conference training session of the 1st Africa Energy Materials (AEM-2017) conference. This enabled maximum participation by non-consortium members and other institutions since participants of the workshop had registered to attend the AEM-2017 conference. The content of the workshop was within the emerging field of materials for energy applications. The workshop focussed on theory, synthesis strategies, characterization techniques and industrial applications. More specifically, the areas that were covered include waste-to-energy, photovoltaics, batteries, hydrogen storage and characterization of energy materials (in particular using X-ray diffraction). Each thematic area was led by a renowned expert in the field. The participants gained relevant technical skills in these areas and developed a more holistic knowledge of energy materials. The workshop included participants from various institutions, predominantly within South Africa but also participants from Botswana, Kenya, Cameroon and Germany.

Following the very successful pre-conference training workshop, the International Workshop on Porous Materials and their Applications (IWPMA) was born. Three IWPMA events were held in 2018, 2019 and 2021. The focus of the workshops was on porous materials including MOFs, zeolites, porous carbons, clays, porous composites and others, The theme also included applications of the materials in gas storage (hydrogen, methane, carbon dioxide, etc.), as well as electrochemical energy applications, catalysis and other related applications and carbon capture and utilization. The third workshop (IWPMA-2021) was held virtually, had 22 speakers from 27 countries and attracted an average concurrent attendance rate of 89 participants per presentation (368 total registered participants). The participants were from the following countries: Netherlands, Canada, USA, UK, Austria, South Africa, France, Kenya, Poland, Cameroon, Lebanon, Libya, Tunisia, Botswana, Uganda, Nigeria, Egypt, Malaysia, China, Germany, Iran, Pakistan, India, Japan, Finland, Kazakhstan and Singapore.

Overall, these workshops were a tremendous achievement. The attendance was equitable with partners from each partner institution attending in addition to other participants from South Africa and many other countries.

#### 3.1.2. Other workshops and trainings

Technical staff members at Maseno University received training on general good laboratory practices, courtesy of the ACBI programme. The main objectives of the training sessions were to: (i) describe the concepts underpinning quality management; (ii) extrapolate these concepts to laboratories; (iii) link these concepts to the relevant ISO standards; and lastly (iv) determine an area to undertake a laboratory quality-improvement project of which we were given the assignment of undertaking an audit of the process of sampling to analysis as it is being carried out in the chemistry laboratory at Maseno University.

**Figure 6.**
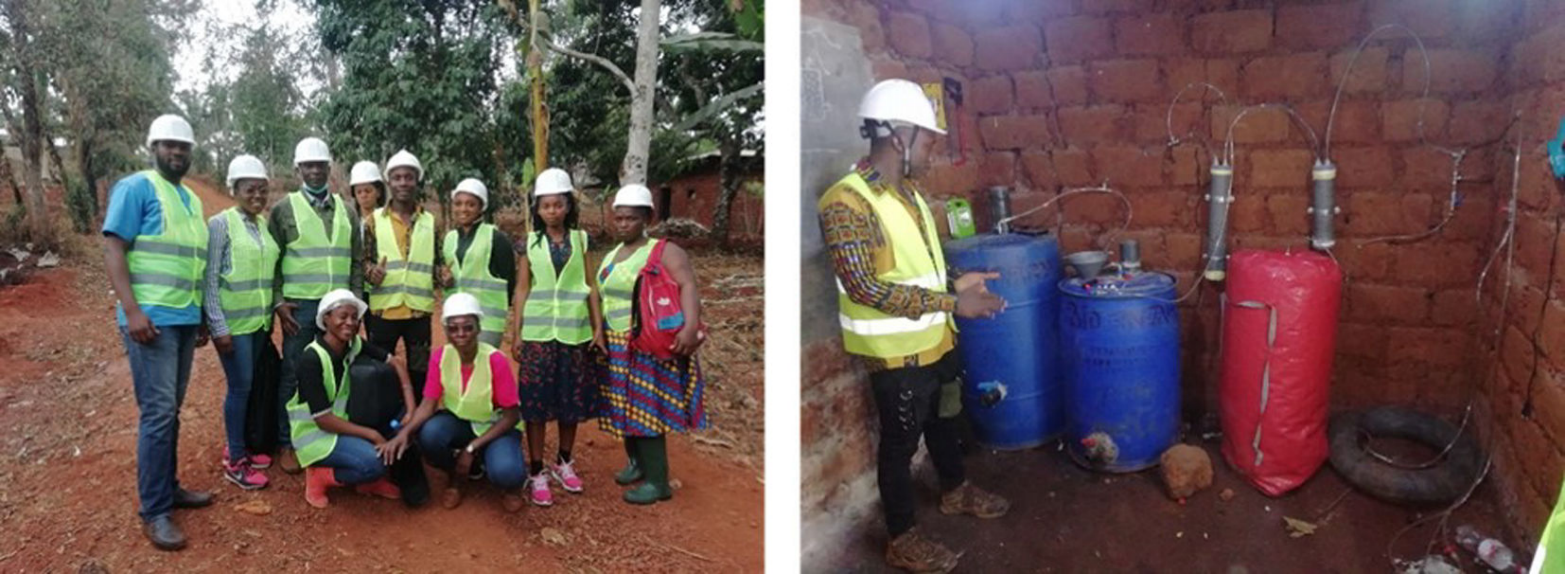
PhD and master’s students supervised by University of Yaounde I PI during field training on biogas production.

In Cameroon, during the COVID-19 pandemic period, two local training workshops aiming to strengthen the capacity of master’s and PhD students in field sampling, paper writing, research questions, mathematical simulation, surface response, data treatment and algorithm writing were held by the University of Yaounde I partner. Furthermore, field training in the production of biogas by a local company in Cameroon specialized in the construction of biogas plants was carried out in 2021 (figure 6) to familiarize the trainees (including master’s and PhD students from University of Yaounde I) with the process, and enable them to explore how research in materials developed in the laboratory can help solve problems encountered in the process of gas production. For instance, problems of gas purification and aqueous waste in which porous functionalized materials synthesized using clay and biomass can be successfully applied.

### 3.2. Glassblowing activities

**Figure 7.**
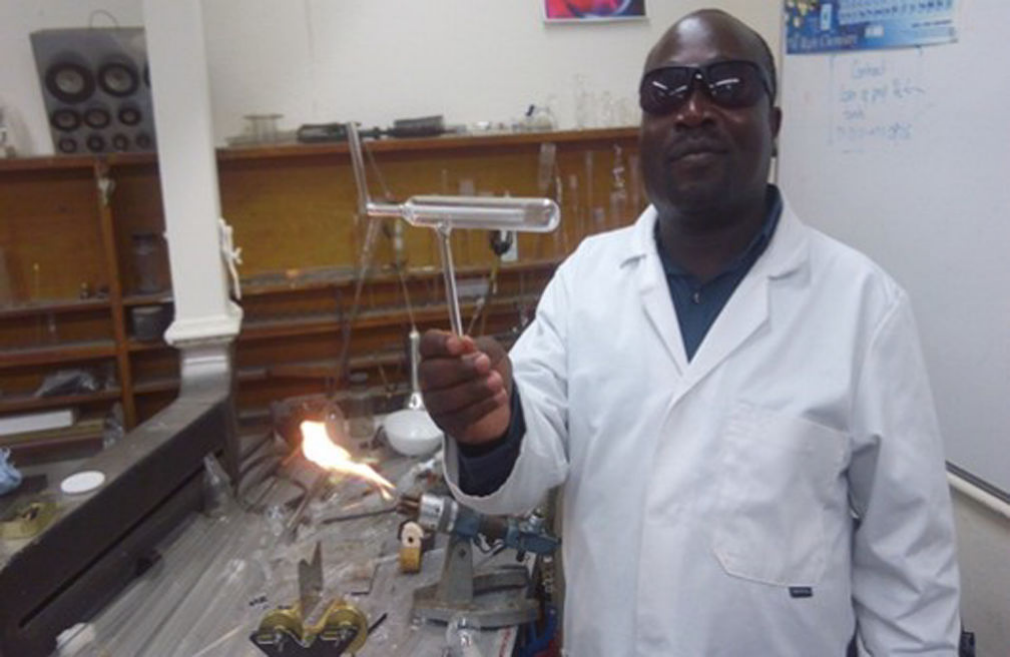
Mr Benard Goga displaying a product of glassblowing.

Ordinarily, glassware consumption and utilization are a great part of laboratory expenses [17]. Maseno University having several laboratories and research activities is therefore no exception to this high consumption of glassware. Capacity building in glassblowing is one way to sustainably manage the glassware shortage in a busy laboratory. In June 2018, a technician at Maseno was identified and supported by the ACBI programme for three months training on glassblowing at the University of Nottingham. For the three months, Mr Bernard Goga (figure 7) was trained practically on the following: cutting glass tubing, pulling spear point, straight joints, unequal joints, T-pieces, Y-pieces, 90^o^ bends, U-bends, button seal, supported internal seals, 1−9 repeated in the medium diameter tubes and bulb blowing. In addition, the trainee had to learn some theories on properties of glass, cutting glass tubing, type of burners, adjustment of burners, types of flames for glass working, safe handling of glasses, and gas cylinders identification.

In addition, training was provided on some analytical techniques including mass spectrometry (MS), gas chromatography (GC), high-performance liquid chromatography (HPLC), nuclear magnetic resonance (NMR), HPLC-MS and GC-MS. The training covered working principles, sample preparation, sample analysis, calibration of these instruments, some of the standards used and their preparation, troubleshooting in case of breakdown during analysis and safety precautions during analysis. In late 2018, a case of glassblowing items was kindly donated by the University of Nottingham to Maseno University. The items were then set up in the Department of Chemistry at Maseno University where they now offer some limited capacity for the repair of smaller-sized glass materials.

### 3.3. Equipment acquisition

**Figure 8.**
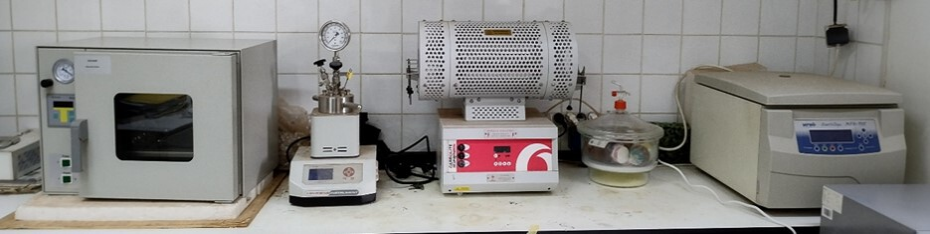
Equipment at the University of Yaounde I, purchased with the ACBI grant.

Strengthening of the research capacity through the Royal Society-FCDO ACBI programme was achieved by the acquisition of several pieces of laboratory equipment at the African institutions. In the case of Yaounde I, from having hardly any equipment in 2016, the following were acquired, thanks to this programme: tubular furnace, two pH meters, analytical balance, centrifuge, vacuum pump, magnetic stirrer, two high-pressure reactors, linear shaker, two potentiostats and stirred microreactor. Some of the equipment is shown in figure 8.

**Figure 9.**
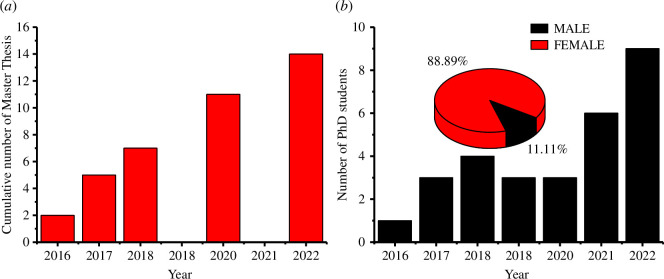
(*a*) Cumulative number of Master’s theses supervised by University of Yaounde IPI and defended during the ACBI programme. (*b*) Evolution of number of PhD studentssupervised by University of Yaounde 1 PI during the ACBI programme. The pie chart insetshows the percentage of students supervised according to gender.

This equipment, in addition to the availability of glassware and analytical grade chemicals, has made the University of Yaounde I research team more attractive with an increased number of master theses defended during the period of the ACBI programme as shown in figure 9*a*. Another beneficial effect of the enhanced research environment is the enrolment and involvement of young women in research as PhD students supervised by the University of Yaounde I Principal Investigator (PI) from 2017 to 2023 (figure 9*b*).

### 3.4. Staff advancement

The Royal Society-FCDO ACBI grant also had a positive impact on staff development, enabling the CSIR PI and co-PI to advance their careers and transition to more senior levels. This progress had a ripple effect on the affiliated students, as evidenced by the numerous awards won. For example, in 2018, the team won the David Sanborn Scott Award from the International Journal of Hydrogen Energy for co-authoring the most cited paper of 2017 in the Hydrogen Storage & Distribution Category. In 2016, the PI received the South African Women in Science Award, earning the title of Distinguished Young Woman Scientist in Physical Engineering Sciences (second runner-up). In 2017, an affiliated student won the best PhD presentation award at the 1st AEM-2017 conference. In 2021, the co-PI won the CSIR Emerging Leader Award and was also awarded the Future Leaders-African Independent Research (FLAIR) Fellowship, a prestigious funding programme for talented African early career researchers funded by the Royal Society in partnership with the African Academy of Sciences (AAS), supported by the Global Challenges Research Fund (GCRF). In addition, the ACBI programme did not only create a favourable research environment but it aided the promotion of the University of Yaounde I PI from Senior Lecturer to Associate Professor in 2017.

### 3.5. Offspring activities

Maseno group has a collaboration with Centre for Innovations and Sustainable Technologies (CIST) Self Help Group in Kisumu town. The CIST group is a self-help community group in the neighbourhood of Maseno University. The youth in the group are making biofuel from water hyacinth through fermentation. The collaboration is exploring diversified sources of feedstock for the process. The bioethanol production process was optimized from aweet sorghum stalk [18,19] and cyanic cassava feedstock. This initiative has helped the group to increase their production capacity from 80 to 250 l d^−1^.

In addition, the indigenous knowledge of biofuel production has attracted interest. Various local societies in Kenya are being interrogated by collecting and documenting the indigenous knowledge and technology on the production of bioethanol. This information will be preserved in a book once sufficient data are available. The idea is to have a scientific understanding of the nature of the traditional raw materials and the characteristics of the yeasts involved. The proposed book title is ‘*Science and practices of indigenous bioethanol productions technology in Africa*’.

At Maseno University, the concepts of energy generation, storage and transport of materials are now embedded into both the MSc and PhD programmes in the Departments of Chemistry and Physics. Some introduction at undergraduate level is also included. The inclusion of energy materials in the teaching curriculum at both undergraduate and postgraduate levels will inspire the next generation of researchers and thus a continuity of the concept initiated by the ABCI programme. The embedding of energy materials in the teaching curriculum at both MSc and PhD levels will imbue both the students and the teaching staff with ideas on energy materials and help sustain the gains of the ACBI programme.

## 4. Conclusion

The research conducted by our consortium in the ACBI programme has made significant contributions to knowledge in fabricating MOFs for hydrogen storage applications. In this regard, we revealed that UiO-66 can be compacted at high pressure leading to improved volumetric hydrogen storage capacity without a trade-off in the gravimetric hydrogen uptake, which is unusual. Furthermore, an uncommon observation of the dynamic behaviour of UiO-66 triggered by the activation temperature was reported experimentally, which led to the proposition of an activation temperature of not more than 150°C for UiO-66. Co-pelletization to fabricate UiO-66/nanofibre monoliths was also demonstrated to be a facile approach for generating hierarchical porous structure in a MOF that was primarily microporous, which is beneficial as it enhanced the usable hydrogen storage capacity of the MOF. Another contribution of our research to scientific knowledge was on the work on zeolites synthesized from naturally occurring kaolin. It was shown that wet mixing prior to dry fusion afforded pure zeolite X with comparable textural properties to those of commercially available zeolite X. In addition, synthesized zeolite X from kaolin was successfully employed as a catalyst to produce biodiesel by transesterification of JC oil, with the optimum catalyst loading being 8%. Our work further led to the preparation of porous material composites (i.e. carbon/UiO-66, organoclay/UiO-66 and zeolite/carbon) that were successfully applied in electrochemical sensing.

Capacity strengthening was an integral part of the ACBI programme and our consortium had success in this regard. The international workshops on porous materials and their applications hosted by the South African partners were a tremendous achievement; the attendance was beyond the four consortium partners and we are happy to have contributed to knowledge expansion, exchange and network building from these workshops. The laboratory equipment purchased has contributed to capacity building within each of the African partner institutions. Notably, the University of Yaounde I PI’s laboratory went from almost no equipment at the start of the ACBI programme to a laboratory now boasting of several functional pieces of equipment, glassware and reagent-grade chemicals. This has positively influenced the research environment attracting master’s and PhD students, especially women, to the research group of the PI. The research exchanges and visits expanded the knowledge base of the students and staff involved in the visitations. There was hosting of PhD students at the University of Nottingham where the students accessed state-of-the-art laboratory facilities, which resulted in highly regarded publications. The ACBI grant contributed to the career development of the PIs and Co-PIs with several awards and promotions granted. The ACBI project has led to a collaboration between Maseno University and a self-help community group that is producing cooking biofuel from water hyacinth. Lastly, due to the ACBI programme, energy materials have been included in the teaching curriculum at both undergraduate and postgraduate levels at Maseno University.

Several outcomes were realized as a result of the unique structure of the ACBI funding. The ACBI funding was able to bring together four institutions that had not previously collaborated on a research project, which culminated in good-quality research outputs and promoted intra-Africa links that would otherwise not have been achieved. The PhD students from the programme gained considerable exposure in an international setting. Another outcome of this collaboration was the training of a technician from Maseno University in glassblowing at the University of Nottingham, a skill that was previously non-existent at Maseno University. The fact that the ACBI funding allowed for the training of technicians enabled this training to be achieved, which is now benefitting not only a specific research group but also the Department of Chemistry and Maseno University at large. This would not have been achieved by the usual unilateral research grants. This structure of the ACBI funding allowed frequent mobility of PhD students, enabling them to spend substantial periods of time (2–4 months) each year at the University of Nottingham PI’s laboratory, which otherwise would not have been possible with other funding schemes that award unilateral research grants. The funding also supported the training of African PIs, which would have otherwise not been accomplished, for instance, University of Yaounde I PI received training in electrochemical energy applications at the University of Witwatersrand in South Africa and the University of Paris Sarclay in France. A further outcome is that continued collaboration has ensued post-ACBI with consortium members. For instance, in collaboration with the University of Nottingham, the CSIR and University of Pretoria are among a 13-member consortium partners who were successful in securing 2022 Horizon Europe funding from the European Union.

In our opinion, the ACBI funding supported our consortium to make a positive impact in terms of scientific contributions and capacity strengthening. Based on our experiences as beneficiaries of the ACBI funding, the following recommendations are made for future capacity-strengthening funding programmes: (i) supporting more than one core PhD student per African partner; and (ii) allowing more flexibility on the restriction on the virement of funds between budget headings.

## Data Availability

This article has no additional data.
